# Subdivision of the neotropical Prisopodinae Brunner von Wattenwyl, 1893 based on features of tarsal attachment pads (Insecta, Phasmatodea)

**DOI:** 10.3897/zookeys.645.10783

**Published:** 2017-01-12

**Authors:** Thies H. Büscher, Stanislav N. Gorb

**Affiliations:** 1 Department of Functional Morphology and Biomechanics, Zoological Institute, Kiel University, Am Botanischen Garten 9, 24118 Kiel, Germany

**Keywords:** Phasmatodea, tarsus, euplantulae, Neotropis, scanning electron microscopy

## Abstract

The euplantulae of species from all five genera of the Prisopodinae Brunner von Wattenwyl, 1893 were examined using scanning electron microscopy with the aim to reveal the significance of attachment pads regarding their phylogenetic relationships. The split into the conventional two sister groups is supported by the two-lobed structure of the euplantulae with a smooth surface in the Prisopodini and a nubby surface microstructure in the Paraprisopodini. The two lineages are well distinguishable by this feature, as well as by the shape of the euplantulae themselves. The functional importance of the attachment pad surface features is discussed.

## Introduction

The Prisopodinae Brunner von Wattenwyl, 1893, which occur exclusively in Central and South America, were erected by Karny (1923) and include various species that were later transferred to other subfamilies. Günther (1953) placed the whole group as a subordinate taxon of the Pseudophasmatinae Rehn, 1904. This view is supported as well by Bradley and Galil (1977) as by more recent phylogenetic analyses (Bradler 2009, Goldberg et al. 2015). Zompro (2004) recovered the Prisopodinae and arranged it to the two remaining tribes Prisopodini Brunner von Wattenwyl 1893, which includes *Damasippus* Stål, 1875, *Dinelytron* Gray, 1835 and *Prisopus* Peletier de Saint Fargeau & Serville, 1828, and Paraprisopodini Zompro, 2004 with *Melophasma* Redtenbacher, 1906 and *Paraprisopus* Redtenbacher, 1906. Bradler (2009) found support for monophyletic Pseudophasmatinae, including the genus *Prisopus* based on distinctive characters of the mouthparts. Goldberg et al. (2015) recovered the species *Melophasma antillarum* (Caudell, 1914) as a member of the Pseudophasmatinae based on molecular data. These findings provide preliminary evidence that the two groups Paraprisopodini and Prisopodini are related to the Pseudophasmatinae. We assume that the former Prisopodinae are a subordinate group within the Pseudophasmatinae, but suppose that the subordinate lineages Prisopodini and Paraprisopodini are sister groups considering their characteristic egg morphology (Zompro 2004).

Various attachment devices have evolved on the tarsi and pretarsi of hexapods (Beutel and Gorb 2001, 2006). As attachment systems underlie adaptations to the substrate and the ecology, their appearance and specific structure reflect aspects of the evolution of the species. The phylogenetic relevance of attachment pads has been previously demonstrated for the Hexapoda in general (Beutel and Gorb 2001, 2006) and with emphasis on the Phasmatodea and Mantophasmatodea (Beutel and Gorb 2006, 2008), as well as for such subgroups as the Dermaptera (Haas and Gorb 2004) and the Plecoptera (Nelson 2009). As hypothesised by Gottardo et al. (2015) the micromorphological surface of the euplantulae might bear phylogenetic relevant features as well. In the present study, the euplantulae of taxa from all five genera included in the Prisopodinae were examined. The general shape of the euplantulae and their surface microstructure were compared with the aim of uncovering relationships between the species included in the corresponding tribes. These characters are discussed with the aim to achieve a more accurate characterisation of the two lineages and to evaluate the monophyly and phylogenetic position of this group.

## Methods

One species per genus has been examined from dried specimens using scanning electron microscopy (SEM). Living animals were anaesthetised with CO_2_ and then decapitated. The right metatarsi were dissected at the level of the tibia and fixated in 2.5% glutaraldehyde in PBS buffer on ice on a shaker for 24 h. To soften and reactivate the attachment pads from the tarsi of dried insects, the legs were cut off, rehydrated in a relaxing chamber for 24 h, and then stored in a 10% solution of lactic acid (Gladun and Gumovsky 2006). The tarsi remained in the solution for 24–48 h and then fixated in 2.5% glutaraldehyde in PBS buffer on ice on a shaker for 24 h. Fixated samples were dehydrated in an ascending alcohol series and critical-point dried. The dried samples were mounted on aluminium stubs and sputter-coated with a 15 nm thick layer of gold-palladium. Specimens were observed in the scanning electron microscope (SEM) Hitachi S4800 (Hitachi High-Technologies Corp., Tokio, Japan) at 7 kV of acceleration voltage. Further species were examined in a stereo microscope to ensure the consistency of the SEM findings. A comprehensive list of the examined specimens is provided in the appendix.

## Results

### Paraprisopodini

Zompro (2004) characterised the species of the Paraprisopodini by their elongated abdomen and the shortening of tegmina and alae in comparison to the Prisopodini as synapomorphies. The two groups, *Melophasma* and *Paraprisopus*, are distributed in Northern South America (Brock et al. 2016).

#### *Melophasma antillarum* (Caudell 1914)

**Figure 1.**
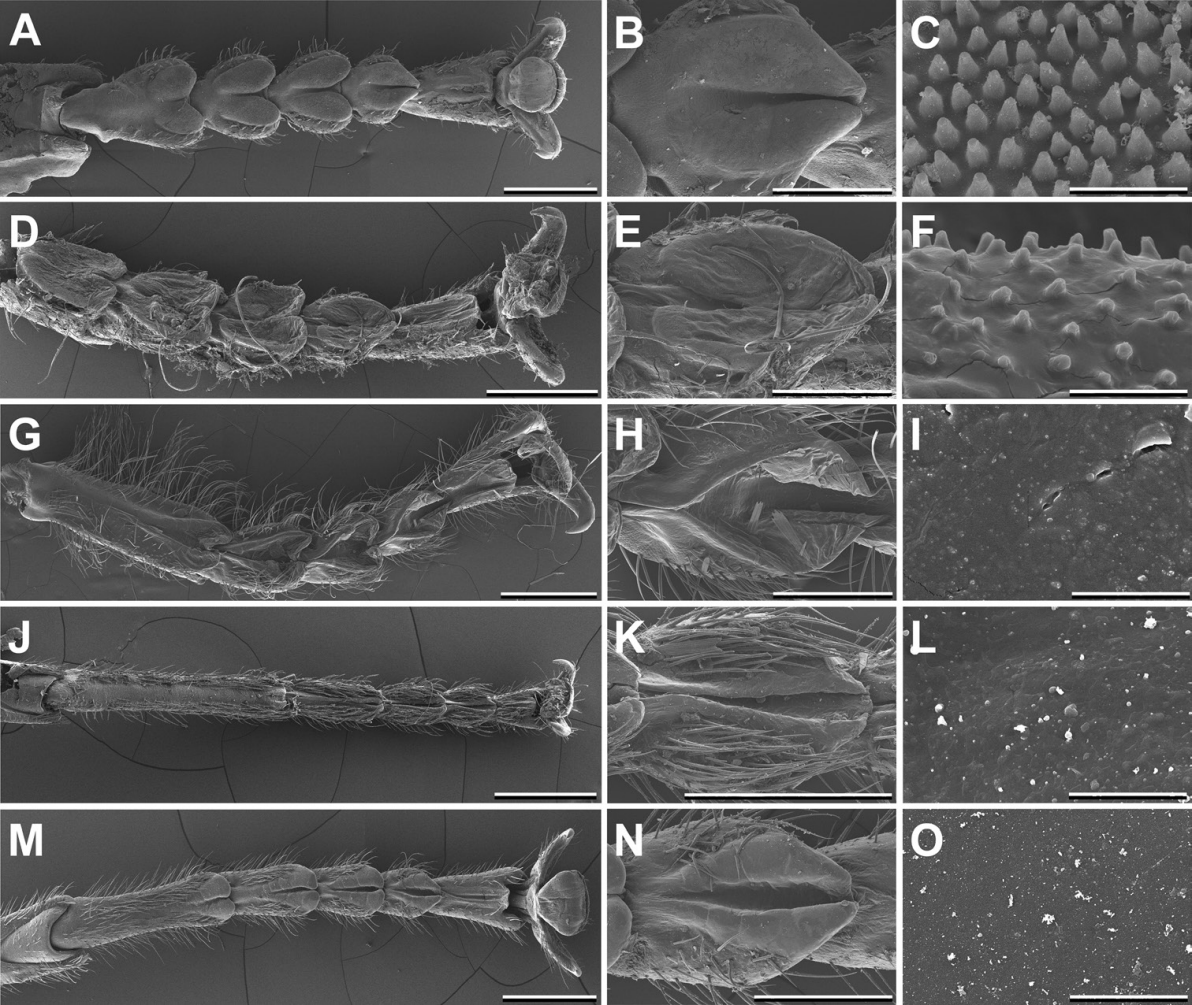
Scanning electron micrographs of the tarsal morphology of different Prisopodinae species. From left to right: Overview; Fourth euplantula; Adhesive microstructure. Scale bars: 1 mm; 300 μm; 5 μm. *Melophasma antillarum*, female (**A–C**). *Paraprisopus merismus*, female (**D–F**). *Prisopus horstokkii*, female (**G–I**). *Dinelytron grylloides*, female (**J–L**). *Damasippus* sp., female (**M–O**).

The tarsi of *Melophasma antillarum* consist of very broad tarsomeres bearing large, roundish euplantulae. The arolium is smaller than the euplantulae. The euplantulae form two separated lobes diverging in lateral direction of the tarsus (Fig. 1A). The flexible adhesive cuticle of the euplantulae is limited to the distal part of the tarsomere forming a clearly cut attachment pad (Fig. 1B). The euplantula surface at high magnification of the SEM reveals small conical outgrowths of the epicuticle (Fig. 1C).

#### *Paraprisopus merismus* (Westwood, 1859)

Similar to *Melophasma antillarum*, the euplantulae of *Paraprisopus merismus* cover a proportionally significant area of the ventral side of tarsomeres, but form hemispherical attachment pads. The arolium is likewise reduced in size (Fig. 1D). All euplantulae form two lobes (Fig. 1E). Although the surface microstructure of the pads consists of similar nubs as in *Melophasma antillarum*, the density of the nubs is lower (Fig. 1F).

### Prisopodini

The representatives of the Prisopodini are also distributed in Central and South America (Brock et al. 2016). This lineage is distinguished from the Paraprisopodini by their long tegmina, which project beyond the second abdominal segment. Additionally, they possess eggs with the operculum inserted dorsally at a positive angle, whereas the eggs of the Paraprisopodini have the operculum inserted at the anterior end (Zompro 2004).

#### *Prisopus horstokkii* (Haan, 1842)

In general the tarsus of *Prisopus horstokkii* has a similar appearance to other Prisopodini, but in detail the tarsal morphology of *Prisopus* reveals unique characters in comparison to the other Prisopodinae. The tarsus is not symmetrical as in the other species, but broadened apically. Additionally, the tarsal setae on the dorsal side of the tarsomeres are much longer in comparison to the other examined genera. The euplantulae of this species are similar to the other Prisopodini, with euplantulae consisting of two bars (Fig. 1G). Each euplantula follows the entire tarsomere length and forms an elliptical pad with a groove (Fig. 1H). In *Prisopus horstokkii*, the surface of the euplantulae is smooth without any microstructural elevations (Fig. 1I).

#### *Dinelytron grylloides* Gray, 1835

In comparison to the tarsi of the Paraprisopodini, the tarsus and the euplantulae of *Dinelytron grylloides* are more slender. Except the first tarsomere, the euplantulae consist of two thin bars traversing the tarsomere and dividing it centrally. The euplantula on the long basitarsus is limited to a small bilobed pad (Fig. 1J). In contrast to the Paraprisopodini and to various other phasmids (Beutel and Gorb 2008), the membranous attachment pad intersects the entire tarsomere (Fig. 1K). The surface microstructure of the euplantulae of *Dinelytron grylloides* is completely smooth in contrast to that of the Paraprisopodini (Fig. 1L).

#### *Damasippus* sp.

The examined specimens are captive-bred from individuals which have been found in Monteverde, Costa Rica and which do not belong to any described species. Similar to *Dinelytron grylloides*, the tarsi of this *Damasippus* sp. individual are slender with a long basitarsus. The euplantulae consist of two bars as well (Fig. 1M). The two bars reach out to each other at the proximal and distal tips, forming an elliptical attachment pad, surrounding a groove in the middle (Fig. 1N). The surface of the euplantulae is smooth, as in the other genera of the tribe (Fig. 1O).

## Discussion

In Zompro (2004), the erection of the Paraprisopodini and the characterisation of the lineages within the Prisopodinae have been done without further discussion of the distinguishing features. The Paraprisopodini are considered by Zompro (2004) the more ‘basal’ tribe of both, because of the shortened tegmina in comparison to the Prisopodini. The tegmina of closely related outgroup taxa (the remaining Pseudophasmatinae) are shorter than of representatives of this group. So the consideration of this shortening as a synapomorphy as brought up by Zompro (2004) is less consistent than considering the elongated tegmina and specialised appearance of the euplantulae in the Prisopodini as synapomorphies.

Due to the shortening of the basitarsus observed in the Paraprisopodini the entire tarsal chain looks shorter than in the Prisopodini. With such geometry, the adhesive force is generated nearer to the body of the insect, which might provide some advantage for controlling attachment and detachment. The Paraprisopodini are camouflaged well in resting position with their legs pulled towards their body. Longer legs on the contrary may be useful for taking longer strides and therefore run faster (Chapman 1998).

All species of the Prisopodinae bear a euplantula on the tarsomere V, which is not the case in all species of the Phasmatodea (Vallotto et al. 2016). Considering the need to attach strongly to the substrate, an additional attachment pad is helpful to increase the attachment force (Gottardo et al. 2015). As the euplantulae are known to generate strong friction (Bußhardt et al. 2012; Labonte and Federle 2013), the use of an additional fifth euplantula might result in a higher total friction force.

The two lineages can be distinguished by the morphological features of the tarsi. The Paraprisopodini bear round, bilobed attachment pads with a nubby adhesive ultrastructure, which correspond to the shape found in many other species of the Phasmatodea (cf. Beutel and Gorb 2008; Vallotto et al. 2016). These two character states are supposed to be a ground plan for the Euphasmatodea (Gottardo et al. 2015) and therefore are suggested to be plesiomorphic regarding the group examined herein. However the arolium being smaller than the euplantulae is a character state not present in the ground plan of the Euphasmatodea, judging on the species covered by the literature (e.g. Beutel and Gorb 2008; Gottardo et al 2015; Vallotto et al. 2016), and might represent an apomorphy on the level of the Paraprisopodini. Bradley and Galil (1977) suggested *Melophasma* being a synonym of *Paraprisopus* without reasoning the synonymisation. This has been ignored by the following publications dealing with *Melophasma*, but is another suggestion of monophyletic Paraprisopodini.

The Prisopodini’s euplantulae on the contrary consist of two thin bars, which are interpreted as an apomorphy of this lineage and support the monophyly of it. Additionally the lack of nubs on the euplantulae is not part of the ground plan in the Euphasmatodea (Gottardo et al. 2015) and is a further potential apomorphy of the Prisopodini. As the euplantulae of the Paraprisopodini match the suggested ground plan of the Euphasmatodea, namely nubby euplantular ultrastructures and roundish bilobed euplantulae, the monophyly of the Prisopodinae sensu Zompro (2004) still has to be validated. Lacking a broader taxon sampling covering closely related outgroups, a synapomorphy of the Prisopodinae sensu Zompro (2004) cannot be found in the attachment devices. A possible synapomorphy of this group is found in the egg morphology, namely a specialised longitudinal area on the ventral surface and the operculum ‘inserted at an angle’ (Zompro 2004). Additionally the tegmina of both lineages are elongated in comparison to the remaining Pseudophasmatinae. Although the tegmina are shorter in the Paraprisopodini in comparison to the Prisopodini, they are of median length regarding the even shorter tegmina of the Pseudophasmatinae.

The examined species of *Damasippus* is found in Costa Rica in dampy and windy habitats. The flying adults are in need of effective attachment organs in order to adhere securely on different substrates when landing, since a fall to the ground would cause troubles to the large animals living up in the tree canopies. The flight of the examined species is not sufficiently effective to return to the foliage without high efforts, but their specialisation to the food plants necessitates a distribution close to them. Considering the slightly concave shape of the euplantulae in this lineage, they may function as a suction cup, generating strong attachment force on rather smooth substrates. It is plausible to assume that, if the bars meet together, they form an ellipse and seal the surrounded volume. The generation of the suction effect can be presumably performed by haemolymph pressure control within the euplantulae (cf. Shvanvich 1949; Dening et al. 2014).

Additionally, both lineages differ significantly in their surface microstructure. So far the nubby surface of the Paraprisopodini is exclusively found in other species with reduced wings. The apterous species *Neohirasea maerens* (Brunner von Wattenwyl, 1907), *Aretaon asperrimus* (Redtenbacher, 1906) (Beutel and Gorb 2008), *Carausius morosus* (Sinéty, 1901) (Bußhardt et al. 2012), and *Conlephasma enigma* Gottardo & Heller, 2012 (Gottardo and Heller 2012) bear nubby surface structures. The species *Orthomeria kangi* Vallotto, Bresseel, Heitzmann & Gottardo, 2016 (Vallotto et al. 2016), *Medauroidea extradentata* (Brunner von Wattenwyl, 1907) (Bußhardt et al. 2012), *Hermarchus leytensis* Zompro, 1997 (Gottardo and Vallotto 2012), and *Eurycantha calcarata* Lucas, 1869 (Gottardo et al. 2015) include both winged and unwinged taxa which all possess smooth attachment structures. Furthermore, pointed acanthae on the euplantulae in *Timema nevadense* Strohecker, 1966 (Gorb and Beutel 2008) and small elevated hexagons in *Dallaiphasma eximius* Gottardo, 2011 (Gottardo 2011) have been previously reported. It is hypothesised that the evolution of different attachment microstructures might reflect phylogenetic patterns (Gottardo et al. 2015). For a proper outgroup comparison of the attachment devices and a more comprehensive comparison of the attachment microstructures a comprehensive study with broader taxon sampling is necessary.

From the functional point of view, smooth phasmid attachment pads demonstrate strong adhesive and frictional performance on smooth substrates, whilst the nubby pad surface seems to be the adaption to a broader range of substrate textures (Bußhardt et al. 2012).

The species *Melophasma antillarum* bears euplantulae, which are known to mainly generate friction, but possesses a reduced arolium, which generates adhesion (Labonte and Federle 2013). The increased role of friction in this species reveals the likeliness of this species to use the tarsi in sliding direction instead of relying on the attachment force directed orthogonally from the ground. In the resting position, representatives of the Paraprisopodini pull their short legs towards their body and rely primarily on friction during attachment. This is reflected in their substrate preferences: they usually occupy thinner branches with small curvature radii. In contrast, individuals from the Prisopodini, which rather prefer flat substrates, rather stretch their legs away from the body, raising the friction contribution due to lowering the angle between the tarsus and the substrate, needed for a proper attachment to the substrate. Together with a comprehensive study of the adhesive structures of the Phasmatodea to evaluate the phylogenetic distribution of these features, we suggest some experimental studies measuring the adhesive properties of different attachment ultrastructures. These may also help to scrutinise the assumptions on the functional morphology of the taxa examined herein.

## Conclusions

Within the Prisopodinae two types of attachment pads are found coherently for the two previously suggested lineages (Paraprisopodini and Prisopodini). It is shown here that characters of attachment pads are useful for distinguishing these lineages. The Paraprisopodini bear big and roundish bilobed euplantulae, as most other known Euphasmatodea, whilst the Prisopodini bear two-bared euplantulae with a groove intersecting the entire tarsomere as an apomorphy. Additionally, the two lineages can be distinguished by the micromorphology of the pad surface. Whilst the Paraprisopodini bear nubby euplantulae with specific densities of nubs, the Prisopodini’s euplantulae are smooth without any micromorphological features. Both macroscopical and microscopical characters contribute to the differentiation of the two lineages, which formerly were distinguished by the tegmina only. The use of the pad surface microstructure for the phylogeny of these groups is suggested in this study for the first time. To validate the monophyly of the former Prisopodinae and their location within the Pseudophasmatinae a more comprehensive study of the attachment ultrastructures of the Phasmatodea in combination with upcoming transcriptome analyses are suggested.

## Appendix 1

### Abbreviations

Coll. TB
 Private collection of Thies Büscher, Kiel, Germany

NHMUK
 Natural History Museum London, UK

NMW
 Natural History Museum Vienna, Austria

OUMNH
 University Museum of Natural History Oxford, UK

ZFMK
 Zoologisches Forschungsinstitut und Museum „Alexander Koenig“, Bonn, Germany

HT
 Holotype

ST
 Syntype

PT
 Paratype

SEM
 Scanning electron microscope

### List of examined specimens

*Damasippus* sp.; coll. TB: 2♂♂, 2♀♀; one female examined via SEM

*Damasippus* sp.; NHMUK: 1♂, 3♀♀

*Damasippus batesianus* (Westwood, 1859); OUMNH, HT: 1♂

*Damasippus discoidalis* Redtenbacher, 1906; ZFMK: 1♂

*Damasippus fuscipes* Redtenbacher, 1906; NHW, ST: 2♂♂, 1♀

*Damasippus fuscipes* Redtenbacher, 1906; NHMUK, ST: 1♂

*Damasippus striatus* Redtenbacher, 1906; OUMNH: 1♀

*Damasippus zymbraeus* (Westwood, 1859); OUMNH, ST: 2♂♂, 1♀

*Damasippus zymbraeus* (Westwood, 1859); OUMNH: 1 nymph

*Dinelytron agrion* Westwood, 1859; NHMUK, HT: 1♂

*Dinelytron agrion* Westwood, 1859; OUMNH: 1♂

*Dinelytron grylloides* Gray, 1835; coll. TB: 1♀; examined via SEM

*Melophasma antillarum* coll. TB: 6♂♂, 6♀♀; one female examined via SEM

*Melophasma vermiculare* Redtenbacher, 1906; NHW, ST: 2♀♀

*Paraprisopus* sp.; NHMUK: 3♂♂

*Paraprisopus merismus* (Westwood, 1859); coll. TB; 1♀; examined via SEM

*Paraprisopus merismus* (Westwood, 1859); NHMUK, HT: 1♂

*Paraprisopus foliculatus* Redtenbacher, 1906; NHW, ST: 1♀

*Prisopus ariadne* Hebard, 1923; NHMUK: 1♀

*Prisopus berosus* Westwood, 1859; NHMUK, ST: 2♂♂

*Prisopus berosus* Westwood, 1859; OUMNH: 1♂

*Prisopus cepus* Westwood, 1859; OUMNH, HT: 1♂

*Prisopus cepus* Westwood, 1859; OUMNH: 2♀♀, 1 nymph

*Prisopus cornutus* Gray, 1835; OUMNH: 1♂

*Prisopus cornutus* Gray, 1835; NHMUK: 1♂

*Prisopus horridus* (Gray, 1835); OUMNH: 1♀

*Prisopus horstokkii* (Haan, 1842); coll. TB: 3♂♂, 1♀; one female examined via SEM

*Prisopus horstokkii* (Haan, 1842); NHMUK: 3♂♂, 2♀♀

*Prisopus phacellus* Westwood, 1859; NHMUK, HT: 1♂

*Prisopus phacellus* Westwood, 1859; NHMUK: 2♂♂, 1♀

*Prisopus phacellus* Westwood, 1859; OUMNH: 2♀♀

*Prisopus sacratus* (Olivier, 1792); OUMNH: 3♂♂, 2♀♀, 2 nymphs

*Prisopus sacratus* (Olivier, 1792); NHMUK: 5♂♂, 4♀♀

*Prisopus sacratus* (Olivier, 1792); ZFMK: 1♂

*Prisopus spiniceps* Burmeister, 1838; OUMNH: 1♀
